# Relationship between fatigue and quality of life and related factors in family caregivers of patients on hemodialysis

**DOI:** 10.1186/s12888-023-04934-2

**Published:** 2023-06-14

**Authors:** Roghayeh Akbari, Zahra Farsi, Seyedeh Azam Sajadi

**Affiliations:** 1 Infectious Diseases and Tropical Medicine Research Center, Health Research Institute, Babol University of Medical Sciences, Babol, Iran; 2 Research and Community Health Departments, Nursing School, Aja University of Medical Sciences, Tehran, Iran; 3 Nursing Management Department, Nursing School, Aja University of Medical Sciences, Tehran, Iran

**Keywords:** Fatigue, Quality of life, Family caregivers, Hemodialysis, Caregiver

## Abstract

**Background:**

The incessant and stressful nature of providing care to patients with chronic diseases can cause fatigue in caregivers. Caregivers’ fatigue and reduced quality of life can reduce the patient’s quality of care. Since it is important to pay attention to the mental health of family caregivers, this study investigated the relationship between fatigue and quality of life and their related factors in family caregivers of patients on hemodialysis.

**Methods:**

This cross-sectional descriptive-analytical study was performed in 2020–2021. One hundred seventy family caregivers were recruited by convenience sampling from two hemodialysis referral centers in the east of Mazandaran province, Iran. The data collection tools were the Family Caregiver Quality of Life questionnaire and Krupp’s fatigue severity scale.

**Results:**

The majority (88%) of caregivers had moderate to severe fatigue. Caregivers’ fatigue was a major factor influencing their quality of life. There was a significant fatigue difference between some categories of kinship and the caregiver’s income level (*P* < 0.05). Caregivers with lower income and education levels, those who were the patient’s spouse, and those who could not leave the patient alone had significantly worse quality of life than other caregivers (*P* < 0.05). Also, caregivers living with the patient in the same house had a worse quality of life than those living separately (*P* = 0.05).

**Conclusion:**

Considering the high prevalence of fatigue among family caregivers of patients on hemodialysis and its adverse effect on their quality of life, it is recommended to perform routine screenings and implement fatigue alleviation interventions for these caregivers.

## Background

With increasing life expectancy in recent decades, many countries, including Iran, have experienced an increased incidence of chronic diseases [1, 2]. Although there are no reliable statistics on the number of caregivers of patients with chronic diseases in Iran, considering the number of these patients and the aging of the population in the country, perhaps a notable part of the Iranian population is likely to provide care to the sick, elderly, and disabled [3]. For patients with chronic kidney failure who undergo hemodialysis, family members have a great impact on how well they manage the disease and their quality of life [4, 5]. It has been shown that the support of family members and close acquaintances improves the survival, treatment compliance, and quality of life of patients with chronic kidney failure [6]. In Iran, individuals are also deeply committed to traditions, and there are strong emotional relationships among family members. This traditional structure is one of the leading support sources for patients [7].

Certain factors distinguish patients on hemodialysis from other patients with chronic diseases. Most importantly, these patients depend on the dialysis machine, which also changes the lives of their family caregivers. The family caregivers of patients on hemodialysis experience disorders such as stress, depression, melancholy, anxiety, irritability, impatience, aggression, fatigue, delirium, and loss of concentration [8]. Evidence showed that the caregivers of patients on hemodialysis experience the same level of anxiety, fatigue, and depression as the patients themselves [9].

Family caregivers are forgotten saviors [3], suffering from the neglect of self-care and receiving inadequate support from the healthcare system. The incessant and often lifelong process of providing care can also cause mental fatigue in caregivers [10]. For example, in one study, 53% of caregivers of patients with cancer reported having moderate to severe fatigue. A significant relationship was found between fatigue and the effect of care on the person’s daily schedule [11].

The main problems facing these caregivers include emotional instabilities and reactions, care fatigue, deterioration of the caregiver’s health, and economic and social issues [12]. Fatigue is an important health indicator which predicts a variety of diseases, more frequent use of health services, and premature mortality. Research into the effect of fatigue on caregivers’ physical health can help identify critical time points and potential targets for interventions [13]. Fatigue is associated with a range of negative outcomes, including poor work performance, negative emotions, and even an increased risk of sudden death [14].

Some have defined fatigue as a complex and non-specific subjective phenomenon that occurs when a person feels that the demands of a process or situation exceed their resources and that there are not enough mechanisms or opportunities for recovering from the pressure. Despite the impact of fatigue on people’s quality of life, few studies have been conducted specifically on the evaluation and treatment of fatigue in caregivers [15].

Family members of patients on hemodialysis are more affected by the treatment in hospitals and therefore have a worse quality of life than other caregivers [16]. A decline in the quality of life of these caregivers can put them under additional pressure and disrupt the care process [17]. In one study, family caregivers with a lower education and income level, who were living in the suburbs, were unemployed, were the patient’s spouse, could not leave the patients alone, were living with the patient in the same house, and were providing care to male patients had significantly worse quality of life than other caregivers of patients on hemodialysis [18]. On the one hand, having good feelings was the primary goal of the World Health Organization in 2001 [19], and improving the quality of life of family caregivers can promote the quality of care provided to patients [20].

Because of the critical role of family caregivers in providing care to patients on hemodialysis, these caregivers experience progressive exhaustion. Thus, more attention needs to be paid to their general health, quality of life, social well-being, and satisfaction. Neglecting the mental state of family caregivers of patients with chronic diseases may also have grave consequences for the patient’s health. Therefore, further research should be conducted on the characteristics of family caregivers and the effects of care burden on their quality of life [20].

The search for reliable databases has shown that although limited research has been conducted on the relationship between fatigue and variables such as depression and quality of life in patients on hemodialysis [21–24], none has investigated fatigue and its relationship with the quality of life of the family caregivers of these patients. Since fatigue and quality of life can be effective in providing continuous and quality care to patients, it is necessary to investigate the severity of fatigue and its relationship with quality of life in family caregivers of patients on hemodialysis.

For the above reasons, the present study aimed to investigate the relationship between fatigue and the quality of life of family caregivers of patients on hemodialysis. The hypothesis of our study was there is a relationship between quality of life and fatigue in family caregivers of patients on hemodialysis.

## Methods

This cross-sectional descriptive-analytical study was conducted from 2020 to 2021 in two hemodialysis referral centers in the east of Mazandaran province, Iran. Both hospitals are in urban areas. But due to the short distance between villages and cities in Mazandaran province, the residents of the villages have access to these two centers. Most go for hemodialysis three times a week (on average, 300 adult patients undergo hemodialysis in these two centers). The appropriate sample size was estimated at 164 subjects (with H_1_ ρ^2^ = 0.1, 1-β = 95%, α = 0.05, and the number of predictors = three) using G-Power software version 3.1.9.2, but considering a 3% dropout probability, 170 eligible subjects were recruited by convenience sampling. Due to the continuous presence of the researcher and the follow-up of the data collection process, and sufficient explanations on how to complete the questionnaires and gain trust, the attrition rate of the sample was zero. In addition, in both centers, dialysis is done only for adults.

The inclusion criteria were the patient undergoing hemodialysis three times a week and the caregiver being older than 18 and being the patient’s family member (in Iran, family members are still the largest group of informal caregivers, and there is a strong commitment and emotional ties between family members [7]), and not having experienced psychological or financial crises, serious diseases, or the death of loved ones based on self-expression during the last six months. The exclusion criterion was the caregiver’s refusal to continue participating in the study.

### Data collection

Data were collected by one researcher by visiting the hemodialysis centers in person. The data collection tools were an individual characteristics questionnaire, the Fatigue Severity Scale (FSS), and the Family Caregiver Quality of Life questionnaire. The data collected by the individual characteristics questionnaire were the patient’s and caregiver’s age and gender, marital status, education level, occupation, kinship, income level, type of insurance, daily hours of care, number of people assisting the main caregiver, and the duration of hemodialysis. The FSS originally developed by Krupp et al. (1989). It measures a person’s perception of their fatigue with 9 items, which are scored from 1 to 7 on a Likert scale. This scale has been used in a great number of studies for fatigue measurement. The total score of this scale is divided by 9, giving a final score ranging from 1 (no fatigue) to 7 (maximum fatigue). A final score of 5 or higher indicates severe fatigue and a score of 2 to 4 indicates moderate fatigue [25–28].

The validity and reliability of FSS have been confirmed in multiple Iranian studies. For example, two studies have reported a Cronbach’s alpha of 0.95 and 0.96 for the internal consistency of its items and an intraclass correlation coefficient of 0.93 and 0.98 for the reliability of its Persian version [29, 30].

The Family Caregiver Quality of Life questionnaire was developed and validated by Sajadi et al. (2018) for family caregivers of patients on hemodialysis. This questionnaire consists of 34 items in 5 dimensions: care burden (items 1 to 15), conflict (items 16 to 21), positive perception of situations (items 22 to 24), self-actualization (items 25 to 28), and fear and concern (items 29 to 34). This questionnaire was designed according to the Consensus-based Standards for the selection of health Measurement Instruments (COSMIN) in the Iranian context. The responses to the items have been scored on a 5-point Likert scale (Completely Disagree; Disagree; Neither Agree nor Disagree; Agree; Completely Agree). A higher total score indicates a better quality of life. The tool’s overall average content validity and Cronbach’s alpha have been 0.89 and 0.90, indicating very good internal consistency and reliability, respectively. The tool’s intraclass correlation coefficient is 0.97, which also indicates good reliability [7].

### Data analysis

The data were analyzed using SPSS version 16. The Kolmogorov-Smirnov test was used to determine whether the data were normally distributed. Then, the data were analyzed by descriptive statistics (mean, SD, frequency, and percentage) and analytic statistics (Spearman and Pearson correlation coefficient, Eta coefficient test, independent *t*-test, one-way ANOVA, and Chi-square test), and multiple regression with the Enter method. In the regression process, all independent variables were entered simultaneously into the model to determine their effect, whether significant or insignificant on caregivers’ quality of life. The multicollinearity was investigated by the variance inflation factor (VIF). The VIF determines the strength of the correlation between the independent variables. The significance level was considered *P* < 0.05.

### Ethical considerations

The ethics committee of Babol University of Medical Sciences (IR.MUBABOL.REC.1399.312) approved this study. Before recruiting the caregivers, they were informed about the study objectives and assured that their participation would be voluntary, they could withdraw from the study at any time, their refusal to participate would not affect the care provided to their patients, and their data would remain confidential. Interested caregivers were asked to provide oral and written informed consent. The study was conducted in compliance with the Declaration of Helsinki and COPE guidelines on publication ethics.

## Results

### Individual characteristics of caregivers

Out of 170 subjects, the majority of caregivers were female (52.9%), married (77.1%), and either the patient’s child (42.4%) or spouse (35.3%). The majority (88.2%) of caregivers had moderate to severe fatigue. Table 1 shows the individual characteristics of caregivers.

**Table 1:** Individual characteristics of family caregivers of hemodialysis patients

Variable	Mean (SD)	Minimum	Maximum
**Caregiver’s age (years)**	45.39 (14.71)	18	78
**Daily hours of care**	11.94 (9)	1	24
**Duration of caregiving (months)**	50.99 (55.19)	0.5	240
**Number of caregivers**	2.69 (1.35)	1	5
**Patient’s age (years)**	57.68 (16.97)	2	96
**Duration of hemodialysis (months)**	41.36 (45.64)	1	228
**Fatigue score**	3.85 (1.70)	1	7
**Total quality of life score**	105.96 (21.49)	54	161
**Care burden**	44.63 (14.87)	16	75
**Conflict**	22.73 (4.91)	6	30
**Positive perception of situations**	10.54 (2.61)	3	15
**Self-actualization**	14.34 (3.63)	4	20
**Fear and concern**	13.72 (4.64)	6	30
**Variable**	**Categories**	**f**	%
**Caregiver’s gender**	Female	90	52.9
	Male	80	47.1
**Marital status**	Single	34	20
	Married	131	77.1
	Divorced	2	1.2
	Widow (er)	3	1.8
**Residence**	Urban	111	65.3
	Rural	59	34.7
**Education level**	Illiterate	30	17.6
	Elementary	23	13.5
	junior-high-school	23	13.5
	diploma	46	27.1
	Associate’s degree	15	8.8
	bachelor’s degree	20	11.8
	master’s & PhD degree	12	7.1
**Occupation**	Housewife	70	41.2
	Self-employed	31	18.2
	Employee	18	10.6
	Worker	13	7.8
	Retired	11	6.5
	Unemployed	6	3.5
	Farmer	12	7.1
	Student	9	5.3
**Income level**	High (sufficient)	28	16.5
	Moderate	76	44.7
	Low (insufficient)	66	38.8
**Insurance**	Social Security Organization	91	53.5
	Medical Services Insurance Organization	32	18.8
	Iran Health Insurance Organization (public health insurance)	7	4.1
	Rural Health Insurance	15	8.8
	Armed Forces Health Insurance	1	0.6
	Special Insurance Schemes	2	1.2
	No insurance	22	12.9
**Kinship**	Spouse	60	35.3
	Child	72	42.4
	Father	6	3.5
	Mother	5	2.9
	Sister	10	5.9
	Brother	9	5.3
	Son/daughter in law	8	4.7
**Living with the patient in the same house (co-occupancy)**	Yes	126	74.1
	No	44	25.9
**Possibility of leaving the patient alone**	Yes	45	26.5
	No	69	40.6
	To some extent	56	32.9
**Patient’s gender**	Female	100	58.8
	Male	70	41.2
**Number of people assisting the main caregiver (in providing care to the patient)**	Zero	38	22.4
	One	48	28.2
	Two	36	21.2
	Three	22	12.9
	More than three	25	14.7
**Fatigue severity**	None	20	11.8
	Moderate	94	55.3
	Severe	56	32.9

SD: standard deviation; f: frequency

### Correlation of caregiver’s fatigue and quality of life with quantitative variables

Factors associated with an increase in fatigue included a longer duration of patient care, higher income, a reduced number of care partners, and low education level (*P* < 0.05) (Table 2).

**Table 2:** Correlation of caregiver’s fatigue and quality of life with quantitative variables

Variable	Fatigue			Quality of Life		
	**Correlation Coefficient**	***P*** **-value**	**Test**	**Correlation Coefficient**	***P*** **-value**	Test
**Caregiver’s age (years)**	0.120	0.122	Pearson	-0.257	*0.001	Pearson
**Daily hours of care**	0.123	0.127	Pearson	-0.234	*0.003	Pearson
**Duration of caregiving (months)**	0.171	*0.030	Pearson	-0.163	*0.039	Pearson
**Number of caregivers**	-0.193	*0.012	Pearson	0.108	0.162	Pearson
**Patient’s age (years)**	-0.072	0.350	Pearson	0.004	0.956	Pearson
**Duration of hemodialysis (months)**	0.130	0.093	Pearson	0.956	0.255	Pearson
**Education level**	-0.189	*0.014	Spearman	0.252	*0.001	Spearman
**Income level**	0.278	0.0001 >*	Spearman	-0.337	0.001 >*	Spearman

*P < 0.05

Factors associated with worse quality of life included older age, more daily hours of care, longer duration of patient care, higher income, and higher education level (*P* < 0.05) (Table 2).

The findings also showed a significant moderate inverse relationship between caregiver fatigue and caregiver quality of life (r=-0.464, *P* < 0.001). (Table 2). In other words, more fatigue was associated with a worse quality of life.

### Correlation of fatigue and quality of life of family caregivers of patients on hemodialysis with qualitative variables

The Eta coefficient showed a weakly significant correlation between caregiver fatigue score and caregiver’s gender, marital status, residence, occupation, insurance type, kinship, living with the patient in the same house (co-occupancy), the possibility of leaving the patient alone, and the patient’s gender (Table 3).

**Table 3:** Correlation of fatigue and quality of life of family caregivers of hemodialysis patients with qualitative variables

Variable	Fatigue	Quality of life
	Eta Coefficient *	Eta Coefficient *
**Caregiver’s gender**	0.046	0.068
**Marital status**	0.105	0.098
**Residence**	0.106	0.016
**Occupation**	0.256	0.216
**Insurance**	0.185	0.166
**Kinship**	0.272	0.327
**Living with the patient in the same house (co-occupancy)**	0.238	0.146
**Possibility of leaving the patient alone**	0.128	0.310
**Patient’s gender**	0.227	0.080

*Chi-square

According to the Eta coefficient, the quality of life score had a weakly significant correlation with the caregiver’s gender, marital status, residence, occupation, insurance type, Living with the patient in the same house (co-occupancy), and the patient’s gender. Findings showed that caregivers’ quality of life scores were moderately related to their relationship with the patient and the possibility of leaving the patient alone (Table 3).

### Fatigue and related factors

The findings showed no significant fatigue difference between the categories of patient’s gender, caregiver’s gender, marital status, residence, occupation, education level, insurance, or the possibility of leaving the patient alone (*P* > 0.05). Also, caregivers living with patients did not have a significantly different fatigue level than other caregivers (*P* = 0.320). However, there was a significant fatigue difference between some categories of kinship and income levels. Caregivers who were the patient’s spouse and those who had lower incomes experienced more fatigue (*P* < 0.05) (Table 4).

**Table 4:** Fatigue and quality of life differences between the categories of individual characteristics of family caregivers of hemodialysis patients

Variable	Category		Fatigue		Quality of Life	
			**Mean (SD)**	**Statistics, df, and** ***P*** **-value**	**Mean (SD)**	Statistics, df, and *P*-value
**Caregiver’s gender**		Female	3.93 (1.75)	*t = 0.601 df = 168 *P* = 0.612	104.58 (22.97)	*t = 0.888 df = 168 *P* = 0.376
		Male	3.77 (1.64)		107.51 (19.73)	
**Marital status**		Single	3.80 (1.47)	**F = 0.1619 d_f =_ 3 *P* = 0.604	108.21 (18.43)	**F = 0.537 df _=_ 3 *P* = 0.65
		Married	3.90 (1.77)		105.05 (22.26)	
		Divorced	3.78 (1.72)		108.50 (19.09)	
		Widow	2.56 (0.80)		118.33 (25.81)	
**Residence**		Urban	3.72 (1.74)	*t=-1.375 df = 168 *P* = 0.171	105.70 (22.75)	*t=-0.212 df = 168 P = 0.832
		Rural	4.10 (1.61)		106.44 (19.08)	
**Education level**		Illiterate	4.50 (1.85)	**F = 1.38 df _=_ 6 *P* = 0.225	92.87 (20.58)	**F = 3.03 df _=_ 6 *P* = 0.008
		Elementary	4 (1.54)		108.13 (22)	
		junior-high-school	4.04 (1.85)		102.39 (19.22)	
		diploma	3.63 (1.72)		109.24 (19.12)	
		Associate’s degree	3.64 (1.45)		108.87 (16.62)	
		bachelor’s degree	3.63 (1.52)		111.35 (24.09)	
		master’s & PhD degree	3.16 (1.59)		115.75 (26.55)	
**Occupation**		Housewife	3.88 (1.74)	**F = 1.62 df _=_ 7 *P* = 0.132	105.11 (23.93)	**F = 1.12 df _=_ 7 *P* = 0.348
		Self-employed	3.23 (1.38)		109.03 (18.83)	
		Employee	4.05 (1.87)		109.39 (23.76)	
		Worker	3.75 (1.79)		108.54 (14.55)	
		Retired	4.85 (1.85)		100.45 (12.84)	
		Unemployed	4.61 (1.63)		88.33 (9.30)	
		Farmer	4.26 (1.53)		102.50 (24.76)	
		Student	3.31 (1.49)		114.44 (20.39)	
**Income level**		High (sufficient)	3.16 (1.49)	**F = 6.69 df _=_ 2 *P* = 0.002	118 (17.83)	**F = 10.87 df _=_ 2 *P* < 0.001
		Moderate	3.63 (1.63)		108.62 (21.005)	
		Low (insufficient)	4.39(1.71)		97.79 (20.54)	
**Insurance**		Social Security Organization	3.65 (1.65)	**F = 0.960 df _=_ 6 *P* = 0.454	108.48 (22.11)	**F = 0.771 df _=_ 6 *P* = 0.594
		Medical Services Insurance Organization	4.22 (1.67)		100.81(16.39)	
		Iran Health Insurance Organization (Public Health Insurance)	3033 (2.004)		98.71(22.45)	
		Rural Health Insurance	4.36 (1.38)		105.40 (12.12)	
		Armed Forces Health Insurance	5.22 (0)		91 (0)	
		Special Insurance schemes	3.28 (1.49)		114 (1.41)	
		No Insurance	3.97 (2.03)		29.68 (6.32)	
**Kinship**		Spouse	4.45 (1.69)	**F = 2.17 df _=_ 6 *P* = 0.048	96.62 (21.77)	**F = 3.25 df _=_ 6 *P* = 0.005
		Father	3.72 (1.48)		107 (19.24)	
		Mother	4.16 (2.08)		113.20 (18.03)	
		Brother	3.59 (1.63)		107.78 (18.50)	
		Sister	3.68 (1.06)		111 (13.26)	
		Child	3.42 (1.75)		111.47 (20.74)	
		Son/daughter in law	3.68 (0.95)		112.75 (23.19)	
**Living with the patient in the same house (co-occupancy)**		Yes	4.09 (1.60)	*t = 3.172 df = 168 *P* = 0.320	104.10 (21.79)	*t = -1.919 df = 168 *P* = 0.057
		No	3.17 (1.80)		111.27 (19.92)	
**Possibility of leaving the patient alone**		Yes	3.49 (1.67)	**F = 1.39 df _=_ 2 *P* = 0.252	116.44 (21.86)	**F = 8.84 df _=_ 2 *P* < 0.001
		No	4 (1.69)		99.97 (19.80)	
		To some extent	3.96 (1.71)		104.91 (20.41)	
**Patient’s gender**		Female	3.53(1.55)	*t = -3.019 df = 168 *P* = 0.074	107.39 (18.85)	*t = 0.989 df = 122.18 *P* = 0.324
		Male	4.31(1.80)		103.91 (24.79)	

*Independent *t*-test ; **One Way-ANOVA Test

### Quality of life and related factors

The results showed no significant quality of life difference between the patient’s gender, caregiver’s gender, marital status, residence, occupation, and insurance. However, caregivers with lower income and education levels, those who were the patient’s spouse, and those who could not leave the patient alone had significantly worse quality of life than other caregivers (*P* < 0.05). Also, caregivers living with the patient in the same house had a worse quality of life than those living in a separate house (*P* = 0.05) (Table 4).

### Major factors influencing caregivers’ quality of life according to the regression model

The findings showed that the fitted regression model was significant (F = 3.380, *P* < 0.001). The coefficient of determination between the variables of “age, gender, marital status, residence, education level, occupation, income level, insurance, kinship, daily hours of care, duration of caregiving, number of caregivers, living with the patient in the same house (co-occupancy) with the patient, the possibility of leaving the patient alone, patient’s age, patient’s gender, duration of hemodialysis, fatigue” and “caregiver’s quality of life” was 0.324. This means that the aforementioned variables explain over 32% of the variance of the “caregiver’s quality of life”.

The variables “income level (*P* = 0.030), insurance (*P* = 0.032), and fatigue level (*P* = 0.002)” were good predictors for “caregiver’s quality of life”. These three variables affect the quality of life of caregivers.

In the next stage, the variables “income level, insurance, and fatigue level” were simultaneously entered into the regression model (F = 19.935, *P* > 0.0001). The coefficient of determination between these variables and “caregiver’s quality of life” was determined to be 0.265, meaning they explain over 26% of the variance of the caregiver’s quality of life.

As shown in Table 5, the confidence interval of the variables income level (*P* = 0.001) and fatigue level (*P* < 0.0001) does not contain 1. Therefore, the fitted model suggests these variables have the most impact on the quality of life of caregivers. Also, VIFs of “income level, insurance, and fatigue level” are almost close to one and variables are not correlated.

**Table 5:** Variables related to family caregiver’s quality of life in the regression model

Variables	B	Standard error (SE)	Beta	t	*P*-value	95% Confidence Interval for B		Collinearity statistics	
						**Lower**	**Upper**	**Tolerance**	**VIF**
Income level	-6.976	2.090	− 0.231	-3.339	0.001	-11.102	-2.851	0.927	1.079
Insurance type	-0.020	0.694	-0.002	-0.029	0.977	-1.390	1.350	0.994	1.006
Fatigue	-5.077	0.875	− 0.402	-5.805	< 0.0001	-6.804	-3.350	0.924	1.082
Constant	141.085	5.314		26.549	< 0.0001	130.593	151.577		

VIF: variance inflation factor

## Discussion

The present study investigated the relationship between fatigue and the quality of life of family caregivers of patients on hemodialysis and their related factors. The majority (88%) of caregivers had moderate to severe fatigue, and their fatigue level had a great impact on their quality of life. In one study, around half of family caregivers of adult intensive care unit survivors had significant fatigue [13]. In another study, nearly 95% of family caregivers of patients with cancer suffered from fatigue [31]. One study reported that 53% of caregivers of patients with cancer had moderate or severe fatigue [11]. In a cross-sectional study, 44% of caregivers of patients who visited a psychiatric emergency department during the COVID-19 pandemic were suffering from fatigue [14].

### Fatigue and related factors

In the present study, there was a significant fatigue difference between some categories of caregiver’s kinship and caregiver’s income level, in the sense that caregivers who were the patient’s spouse and those who had lower incomes were suffering from more fatigue. The findings also showed a longer duration of patient care, higher income, a reduced number of care partners, and a low education level were associated with an increase in fatigue.

A study by Talebi et al. also reported that caregivers of patients on hemodialysis who were the patient’s spouses and had taken care of the patient for a longer time, and had lower income and education were under more caregiving burden [32]. According to some studies, fatigue is more common in lower socioeconomic strata. For example, there is evidence showing a higher prevalence of fatigue among people of lower socioeconomic status in South Korea and minimal fatigue in the affluent section of the French population [33]. In one study, caregivers of patients on hemodialysis with a better economic status had lower caregiving burdens [32, 34].

The findings of this study showed no significant fatigue difference between the categories of the patient’s gender, marital status, residence, occupation, insurance, the possibility of leaving the patient alone, and education level. A study on caregivers of patients with cancer also found no significant relationship between the caregiver’s fatigue severity and the caregiver’s age, the patient’s age, and the caregiver’s occupation [11]. But another study reported observing more fatigue in female family caregivers than in male family caregivers [35]. In one study, the fatigue of caregivers of patients who visited a psychiatric emergency department during the COVID-19 pandemic was positively related to higher education levels [14]. The reason for these discrepancies could be the differences in the study populations. Considering the few studies conducted on the subject of fatigue among family caregivers, there were not enough references to further compare the results.

### Quality of life and related factors

The findings showed that there was a significant fatigue difference between some categories. Caregivers with lower income and education levels, those who were the patient’s spouse, and those who could not leave the patient alone had significantly worse quality of life than other caregivers. Also, caregivers living with the patient in the same home had a worse quality of life than those living beside the patient. The findings also showed that older age, more daily hours of care, and a longer duration of patient care were associated with worse quality of life.

A systematic review reported that there is a significant correlation between the quality of life of caregivers of patients with breast cancer and their income level [36]. In a study by Sajadi et al. on the caregivers of patients on hemodialysis, caregivers living in the suburbs, who were unemployed, were the patient’s spouse, could not leave the patients alone, were living with the patient in the same house, provided care to male patients, and had a lower education and income level had significantly worse quality of life than other caregivers [18]. In multiple studies on the caregivers of patients with dementia, caregivers who were the patient’s spouse had a far lower quality of life than those who were the patient’s child [37, 38]. In one study on these caregivers, those living with the patient had a worse quality of life than those living in a separate house [37]. There is also evidence showing a relationship between the duration of dementia and the quality of life of caregivers [39, 40]. In a study on the spouses providing care to veterans with chronic spinal cord injuries, the results showed a significant negative relationship between the caregiver’s age and duration of caregiving and the caregiver’s quality of life in the physical functioning domain, and a direct relationship between the caregiver’s education and their quality of life in this domain [41].

In the present study, the findings showed no significant quality of life difference between the categories of patient’s gender, caregiver’s gender, marital status, residence, occupation, and insurance. In a similar study, there was no relationship between the care burden of family caregivers of patients on hemodialysis and patient’s gender and the patient’s gender or the caregiver’s gender, residence, or occupation [32].

### Relationship between fatigue and quality of life

The findings showed a significant inverse relationship between caregiver fatigue and caregiver quality of life and that fatigue is a major factor influencing caregiver quality of life. In other words, caregivers who were more fatigued reported a worse quality of life.

This shows the confirmation of our hypothesis. In a study on the quality of life of caregivers of patients with end-stage kidney disease undergoing dialysis compared to comprehensive conservative care, the results showed that the vitality domain, representing energy and fatigue is a key driver of the quality of life of caregivers of both groups of patients (undergoing dialysis and conservative care) [42]. In the cross-sectional study on the caregivers of patients who visited a psychiatric emergency department during the COVID-19 pandemic, fatigued caregivers had a significantly worse quality of life than non-fatigued caregivers [14]. In a study conducted on Korean nurses, there was an inverse correlation between acute and chronic fatigue and quality of life [43], which follows the findings of the present study.

### Strengths and limitations

One of the strong points of this study was the use of a valid and reliable local tool for measuring the quality of life of family caregivers of patients on hemodialysis and a standard fatigue measurement tool. In the present study, we did not assess covariates including intrinsic factors related to the caregivers’ quality of life (e.g., personality traits, coping strategies, mental health status, level of patients, and caring capability). The association between the study variables may be attenuated by considering these covariates. And it is crucial to emphasize that cross-sectional studies cannot guarantee accurate predictions. Instead, developing prediction models based on a cohort study is suggested.

## Conclusion

In the present study, caregivers’ fatigue was a major factor influencing their quality of life. Mental health policymakers and health care providers can consider our findings as a basis for the need to create strategies that reduce fatigue and improve quality of life, as well as periodic monitoring of their physical and mental health status to empower family caregivers. Since improving the quality of life of family caregivers ultimately improves the quality of care they provide to patients, it is recommended to implement training programs and fatigue alleviation interventions for these caregivers. Considering the adverse effect of fatigue on quality of life and other health outcomes, it is also recommended to perform routine screenings as the basis for subsequent fatigue interventions. Since some individual characteristics of caregivers were found to affect their quality of life and fatigue, more attention should be directed toward especially vulnerable groups. Other researchers are recommended to conduct further studies on the assessment of fatigue severity among caregivers of patients on peritoneal dialysis or kidney transplantation. Future studies are also recommended to investigate the quality of life and fatigue of patients and family caregivers simultaneously.

## Data Availability

The datasets used and analyzed during the present study are available from the corresponding author upon reasonable request.

## Abbreviations

FSS: Fatigue Severity Scale
